# Assembling care: How nurses organise care in uncharted territory and in times of pandemic

**DOI:** 10.1111/1467-9566.13508

**Published:** 2022-08-05

**Authors:** Syb Kuijper, Martijn Felder, Roland Bal, Iris Wallenburg

**Affiliations:** ^1^ Erasmus School of Health Policy and Management Erasmus University Rotterdam The Netherlands

**Keywords:** assemblage, COVID‐19, ethnography, materialities of care, nursing, organising work

## Abstract

This article draws on ethnographic research to conceptualise how nurses mobilise assemblages of caring to organise and deliver COVID care; particularly so by reorganising organisational infrastructures and practices of safe and good care. Based on participatory observations, interviews and nurse diaries, all collected during the early phase of the pandemic, the research shows how the organising work of nurses unfolds at different health‐care layers: in the daily care for patients and their families, in the coordination of care in and between hospitals, and at the level of the health‐care system. These findings contrast with the dominant pandemic‐image of nurses as ‘heroes at the bedside’, which fosters the classic and microlevel view of nursing and leaves the broader contribution of nurses to the pandemic unaddressed. Theoretically, the study adds to the literature on translational mobilisation and assemblage theory by focussing on the layered and often invisible organising work of nurses in health care.

## INTRODUCTION

The decision was made—we had to establish a cohort unit. Well, when you put these ideas down on paper and have it all figured out, it seems to work fine. But the moment they *[the board]* said “Go for it!”, well, I can still see the look in the eyes of the physician who thought, “Okay then….” *[clueless].* But also, the look in the eyes of the chief nurse, like “Okay!” *[decisive].* The chief nurse just took care of it, and in a blink of an eye, it was all set: the COVID unit was set up, everyone knew what to do, and the materials had been delivered. And the appreciation of the physician, who hadn’t known what to do…To me, that moment revealed a huge difference in expertise. The physician might know everything about the disease, about the treatment, and what is known at that time. But organising care for a patient is a different profession, a different and true craft I’d say.

(Nurse and secretary of the board of directors, February 2021)

In March 2020, the World Health Organization (WHO) declared the outbreak of the novel COVID‐19 virus a global pandemic (WHO, 2020). A few days later, the Dutch prime minister spoke to the nation in a rare, televised address: ‘Many people will recognise the feeling that we have been on a roller coaster in recent weeks that seems to be going faster and faster. You wonder, is this really happening?’ Soon, the pandemic began to strain health‐care systems and put immense pressure on health‐care workers in The Netherlands, as elsewhere in the world (Bal et al., 2020). Before the outbreak, the WHO had designated 2020 as the ‘Year of the Nurse and the Midwife’ to mark nurses’ essential work in challenging conditions. ‘How prophetic that goal turned out to be’, commented the director of a pharmaceutical company later that year (Johnson & Johnson, 2020). Since the surge of the virus, nurses have been at the forefront of organising and delivering COVID care and have come to symbolise the COVID‐19 response (Bennett et al., 2020; Mohammed et al., 2021; Różyk‐Myrta et al., 2021).

The Dutch and international media have displayed a rather one‐dimensional understanding of nursing work that portrays nurses as ‘heroes at the bedside’, fostering ‘classic’ images of nursing work as a primarily passive and compassionate care‐giving and feminised occupation (Bennett et al., 2020; Hennekam et al., 2020; Mohammed et al., 2021; Stokes‐Parish et al., 2020). This image ignores other dimensions of nursing, such as the organising work performed to increase patient capacity and enabling care delivery in times of crisis—as we will show in this article. Moreover, scholars argue that a one‐sided hero discourse may even serve to legitimise and normalise the dangers nurses face and how these dangers are, in turn, used by politicians to legitimise restrictive COVID measures in mainstream and social media (Cox, 2020; Einboden, 2020; Mohammed et al., 2021). Nurses, however, have challenged these professional stereotypes by using the same (social) media platforms to promote the different and skilled aspects of their (crisis) work (Croft & Chauhan, 2021)—underscoring that a one‐sided hero discourse blurs our view of the role that nurses really play in health crisis containment and management.

From a sociological perspective, the emphasis on care and the invisibility of nurses’ organising work are hardly surprising. Scholars have long pointed out how nurses’ organisational competencies are essential to health‐care systems but an invisible and even a contested part of nursing work at the same time (Allen, 2014; van Ewijk, 2013). In our research, we noted that it was often nurses who re‐invented and organised hospitals and health‐care delivery in the response to COVID‐19—as illustrated in our opening quote. In our observations and interviews, we furthermore saw how the organising work of nurses involves highly situated experimentation and emergent decision‐making under critical uncertainty, and how nurses enacted different social and material entities to continue and support health‐care delivery. In this article, we use the momentum of the pandemic to articulate how nurses organise and reorganise care at different organisational levels, both within health‐care organisations and across the health‐care system. In doing so, we seek to go beyond the current and rather normative framing of nursing work and envision how nurses give shape to, and care for, health‐care delivery during a pandemic crisis.

Our aim in this article is to contribute to an emerging sociological literature on the organising work of nurses (e.g., Allen, 2014) by illuminating how nurses mobilise, navigate and adjust sociotechnical assemblages to deliver and organise COVID care. The following research question guides our analysis: *How do nurses mobilise and (re)assemble care in the Dutch COVID‐19 response, and how does this organising work unfold at different organisational levels in times of crisis?*

We draw on insights from the fields of Science and Technology Studies (STS) and Sociology of Health and Illness (SHI) to conceptualise how nurses bring together and mobilise sociotechnical assemblages in organising and delivering COVID care. The concept of assemblage allows us to include human and non‐human actors in the analysis and to study empirically how nurses assemble dispersed entities (e.g., the built environment, technologies, concepts and people) so that they work together and mesh in various temporal orders to provide care (Delanda, 2016; Muller, 2015; Nail, 2017; Sager & Zuiderent‐Jerak, 2020). In our analysis, we build on discussions in assemblage literature that have foregrounded the role of the material and the architectural in health care, and how this relates to the notions of affect, care and normative work (Brown et al., 2020; Buse et al., 2018; Driessen, 2020; Latimer, 2018). As we will show, the COVID‐19 pandemic encompasses various individual and collective crises and related values and goals. In this article, we scrutinise how these different ‘goods’ and ‘bads’ are mediated through nurses’ work and inform the ‘doings’ of COVID care in daily health‐care practice. In the following, we first present the theoretical framework in which we connect assemblage literature to notions of (good) care. We then describe the research methods, followed by a results section in which we show how nurses mediate and move between different layers of the health‐care system and organise and assemble or reassemble care at different levels. We end with a discussion in which we reflect on the implications of our findings and how they contribute to a broader sociological understanding of nursing work.

## ASSEMBLING NURSING CARE

Nursing is traditionally seen as a care‐giving occupation (Gillett, 2012; Yam, 2004), closely connected with ideas of nurturing and even ‘mothering’ (Davies, 2003). Although the image of nursing has been discussed at length in the sociological literature, the profession continues to be characterised and evaluated by this somewhat one‐dimensional care‐giving image, which moreover continues to drive perceptions of nurses as well as their self‐image and hinders the construction of nurses, add, desired professional identity and nursing leadership (Croft et al., 2015). STS literature addressing nursing practices stresses the invisibility of nurses’ organising work as both integral and essential to nursing, and how this invisibility downplays the importance of nursing work and hence the position of the nursing profession. In early work, Timmermans et al. (1998) have shown that making nursing work visible (i.e., through the categorisation and evaluation of their work) makes nurses prone to managerial control. They show that such visibility may hamper instead of improving the autonomous position of nurses and hence recognition of their work, because categorisation and evaluation often rest on traditional notions of care work, stressing nurses’ hands‐on work at the patient’s bedside and overlooking the highly skilled and knowledgeable activities they undertake to enable smooth care delivery across various professional and organisational levels.

More recently, authors have foregrounded the organising work of nurses in the sociological literature on health and illness. For example, Allen’s (2014) ethnographic study of nurses’ daily organisational work and practices has laid a theoretical and empirical foundation for conceptualising how such organising work unfolds in evolving and complex health‐care settings. Allen (2014) illuminates how nurses shape individual patient care trajectories and bring together fragmented care processes by aligning constellations of both human and non‐human actors across organisational boundaries and therefore mobilise and sustain sociotechnical networks in which patient care trajectories unfold. Allen (2014) coins the concept of ‘translational mobilisation’ to point out how nurses validate and interpret dispersed information and thereby piece together trajectory narratives, mediate relationships between various health‐care workers and use their understanding of patients’ psychosocial circumstances and intimate organisational knowledge to perform bed management and care transfers.

The concept of translational mobilisation makes it possible to articulate and understand nurses’ emergent organising work. Doing so reveals the highly skilled and knowledgeable activities nurses undertake to enable health‐care delivery. This literature lays the groundwork for rethinking what nursing work entails and how different forms of expertise and care work are necessary to organise and deliver care in dynamic health‐care settings (Allen, 2019). Emphasising the coordination and planning of care for individual patients or ‘ward care’, however, ignores the broader work nurses do to keep the health‐care system on track, particularly in a pandemic.

To envision this broader contribution of nurses and nursing work, we turn to assemblage literature (Delanda, 2016; Guattari & Deleuze, 1988). This literature allows us to articulate how nurses rebuild and organise hospital departments, reinvent and coordinate working routines and methods, perform critical roles in patient allocation between hospitals and thereby shape care beyond hospital walls and across organisations.

### Nursing work as an assemblage of care

Félix Guattari and Gilles Deleuze (1988) coined the notion of ‘assemblage’ as a theoretical concept and analytical tool. Assemblage literature analyses the social world as constantly in flux. It considers the interactions of various sociotechnical entities and how they come together in temporal and relational formations (Amironesei & Bialecki, 2017; Delanda, 2016). In line with this literature, health‐care organisations can be understood as adaptive, heterogeneous and dynamic organisations in which the role of both human and non‐human actors is articulated. Assembling is therefore about bringing and holding together heterogeneous elements and shows the work needed to make this happen (Ivanova et al., 2016). Assemblage literature sensitises us to how nurses align, adjust and tinker with social and material objects in the COVID‐19 response.

Importantly, assemblage literature has envisioned the role of materiality and the built environment in health‐care practice (Brown et al., 2020; Brownlie & Spandler, 2018; Buse et al., 2018; Heath et al., 2018). In this body of scholarly work, both ordinary and innovative materialities act as a lens to (re)examine the intersection of care, things and architecture (Buse et al., 2018). Heath et al. (2018), for instance, examine in detail how nurses in an operating theatre act on a diverse set of knowledges (e.g., clinical, practical, organizational) to manage and configure various objects used and exchanged during surgical procedures—such as swaps, hammers, drills and scissors—highlighting the role of objects and artefacts in health‐care practice and nurses’ technical and organisational sensitivities. Brown et al. (2020), in their turn, stress the socio‐materialities of care in a respiratory clinic for patients diagnosed with Cystic Fibrosis (CF), illuminating the dangers of airborne (and life‐threatening) virus transmissions in the case of CF patients and the role of nurses and patients in reducing those dangers through social‐material and situated solutions—something remarkably relevant to the current COVID‐19 pandemic. The authors show that good air hygiene and careful aerographic management are critical to lung infections. They describe how patients’ bodies are envisioned as surrounded by a ‘cloud’ of transmissible bioaerosols, rendering air visible, and how this spurs health‐care professionals to organise or reorganise and reshape the built environment to enhance ventilation and minimise gatherings of patients. Thus, instead of seeing materialities as static, they reveal how both nurses and patients—separately but also in their mutual interactions—reimagine and materialise socio‐material entities (e.g., windows, fresh‐air balconies) to make them manageable in everyday care practices, and hence how care for the environment is part of the assemblage of organising CF care. Hence, the focus on this body of literature draws attention to how socio‐material assemblages are managed and configured through nurses organising work.

### Good crisis care

Assemblages include the notions of care work, affect and ‘doing good’ (Ivanova, 2020). According to Tronto (1993), care ‘includes everything that we do to maintain, continue, and repair our world so that we can live in it as well as possible. That world includes our bodies, ourselves, and our environment, all of which we seek to interweave in a complex, life‐sustaining web’ (Tronto, 1993, p. 103). Some scholars have made visible how actors, who are themselves embedded in the sociotechnical assemblages of care, seek to bring or keep values together in their daily practices (de La Bellacasa, 2013; Mol et al., 2010). For instance, Jeannette Pols (2006) shows that ‘good washing’ may sometimes involve not washing patients to respect their autonomy, even when they smell, but at other times forcing patients to shower when their body odour is a cause of loneliness they suffer from. In line with assemblage literature, care is thus described as something to do, as a form of attentive, situated and ongoing tinkering in dynamic entanglements with space, values, materialities and technologies (Driessen, 2020; Ivanova et al., 2016; Martin et al., 2015). These authors also emphasise that assemblages themselves are, by nature, normative (e.g., ‘well as possible’, ‘good care’) and require a constant weighing of what matters, what should and could be done and what should be included and tied together. Hence, the focus on assemblage allows one to articulate how different values and perspectives of ‘good care’ come into play in nurses’ daily care work (Latimer, 2018).

The need to mediate different values became very apparent in the COVID‐19 crisis, when hospitals overflowed with severely ill patients and health‐care professionals needed to tinker with both collective and individual patient needs (de Graaff et al., 2021). These different needs and values may conflict and require negotiation and mediation, in other words being ‘tinkered with’ (Mol et al., 2010). In the following sections, we analyse how different values of and perspectives on good care were enacted and navigated in the daily organising work of nurses during the pandemic, and what this teaches us about the role of nurses’ organising work in health care.

## METHODS

This article builds on an extensive data set stemming from the ‘RN2Blend’ research programme. It is a national research programme that studies the differentiation of nursing jobs and roles in Dutch hospital organisations and University Medical Centers. Nurses’ role in the pandemic is a ‘celebrated’ and topical case that offers a new, sociological perspective from which to study nursing work and professionalisation. Ethical approval for the study was obtained through the internal review board of the Erasmus School of Health Policy and Management.

Initially, early in the pandemic, the constraints of social distancing and other COVID measures complicated in‐person data collection. To capture the experiences and work of nurses in COVID care, we set up a qualitative diary study in April 2020. We asked participating nurses to reflect, in writing or in recorded audio or video clips, on their daily work in COVID care.

Although the study produced some valuable diaries and reflections, it was clear that keeping a diary was burdensome for nurses in times of crisis. Moreover, the diaries we did receive addressed a plethora of topics (e.g., physically restructuring hospital wards, establishing and developing new care routines, decision‐making in critical uncertainty) that we wanted to explore in greater depth. We therefore turned to other, online, research methods and interviewed the initial diary study participants virtually. These semi‐structured interviews (*N* = 27) with frontline staff were conducted from April 2020 to March 2021. All interviews started with open‐ended questions about daily care work in the pandemic. Informed by the initial diary study, we then enquired into the disruptions of clinical routines and teamwork, and decision‐making in the face of uncertainty. We furthermore asked participants to reflect on the quality and safety of care and how nurses contributed to the organisation of care delivery. We recruited nurses working in different roles at various teaching and general hospitals, as shown in Table 1, to capture a wide range of experiences and stories. Although online research methods present limitations and challenges compared to in situ research (e.g., socialising with respondents, taking sensory experiences into account) (Podjed, 2021), digital technologies allowed us to gather rare and valuable insights about day‐to‐day nursing during the first wave of the pandemic. Verbatim transcripts were made of each interview.

**TABLE 1:** Details of interviewed nurses

Type of hospital	Nurse role
Academic	COVID coordinator (4), nurse & secretary of the board of directors (1), IC nurse (3), specialised nurse (2)
General	COVID coordinator (3), unit manager (3), specialised nurse (4), IC nurse (2), registered nurse (5)

Soon after the first wave of the crisis, we continued our research by conducting ethnographic fieldwork in two Dutch hospitals and at the National Centre for the Spread of Patients (LCPS). From November 2020 to February 2021, the first author shadowed three nurse coordinators, as shown in Table 2, as they went about their everyday work in COVID care. We spent 6 days (over 48 h in total) observing nursing coordinators’ daily work and meetings. In addition, in February 2021, we observed several other health‐care professionals, including nurses, working on patient allocation at the LCPS for 3 days (over 24 h in total). Ethnographic research proved valuable for an in‐depth understanding of the daily practices of COVID care and for observing how nurses shape and perform care in times of crisis. The participatory observations enabled us to unravel themes drawn from the diary study and interviews and how the organising work of nurses (e.g., translational and repair work) unfolded in daily practice. Fieldnotes and informal conversations were written up into observational reports within 24 h upon leaving the field. In addition, we held formal interviews with the participants (*N* = 8) that were also transcribed verbatim.

**TABLE 2:** Details of fieldwork

Field work	Methods and respondents
General hospital (2)	*Observations* (48 h), nurse COVID coordinators (3)
National Centre for the Spread of Patients (‘LCPS’)	*Observations* (24 h), health‐care professionals, including nurses
	*Interviews* (*N* = 8), nurse (1), chief medical officer (1), managers (4), mathematic modelers (2)

The data we gathered was analysed abductively (Tavory & Timmermans, 2014), allowing us to make several rounds of iterations between our data and theory (as described in the theoretical section above). Throughout the data analysis process, an iterative and reflexive approach enabled us to explore empirical findings in‐depth.

We started coding based on earlier theoretical insights on nurses organising work (e.g., coordination, translational and articulation work) and different valuations that shape nursing work in the pandemic (e.g., safe, humane, timely and efficient care).

Throughout data analysis, coding yielded new insights that we then investigated and member‐checked with nurses in the field in formal and informal conversations and interviews. Based on what we found within the data, we developed new codes to capture our findings (e.g., organisation and establishment of [COVID‐19] wards, coordination of socio‐materialities across organisational boundaries and caring repair work). Coding was primarily carried out by the first author and then discussed among all co‐authors. All quotes and excerpts are translated from Dutch. In our analysis, we employ pseudonyms to maintain anonymity.

In the following section, we show quotations which are selected to reflect how nurses actively (re)configure social technical assemblages and to elucidate how this organising work of nurses unfolds at three layers: on the ‘shop floor’, in and across hospitals and at the level of the health‐care system.

## RESULTS

It was a Wednesday. I remember clearly that it started abruptly with patients arriving in the emergency department. I helped that day when we moved our entire department on the spot and converted it into a cohort unit.

(Nurse, June 2020)

During the initial outbreak of the novel COVID‐19 virus, health‐care provision had to change overnight. In the above quote, the nurse in question recalls how she and her colleagues were asked to abandon their usual work and help clear and transform specialised nursing wards into provisional cohort units. All of a sudden, this nurse found herself at the forefront of the Dutch COVID crisis response. The following three sections describe how nurses assemble and reassemble care in this new situation, and how this organising work unfolds at different organisational levels. First, we demonstrate how nurses interact with and mobilise materialities, things and technologies while rebuilding and reassembling wards. The second section discusses how nurses’ organising work occurs across organisational boundaries and involves decision‐making under critical uncertainty. In the third section, we work out these findings in more detail by demonstrating how nurses perform patient allocation, thereby contributing to the broader organisation and health‐care system ‘in crisis’.

### Reorganisation of hospital spaces of care

We had two wards. One of them was a COVID ward. That ward was cordoned off with tarpaulin, and the entrance closed off with zippers. When you went in, you had to wear a protective suit, and that’s how we separated the “dirty side” from the “clean side”. That was also when we brought in the walkie‐talkies. It allowed nurses on the dirty side to quickly ask nurses on the clean side to fetch something from the medicine room. Or when a “clean” patient turned out to be infected, we used the walkie‐talkies to inform nurses on the other side, after which the patient could go past the tarp. It made it possible to coordinate between the *[physically separated]* wards.

(Nurse, July 2020)

At the beginning of the pandemic, COVID care was unchartered territory, with no scenario available that described how to treat infected patients. The reorganisation of care involved a considerable amount of experimentation and improvisation. As the exact mechanism of transmission was still unknown, treatment involved exercising immense caution in the face of possible viral transmission through direct contact, aerosols and droplets. This required careful aerographic management and the ability ‘to envision the invisible’ (Brown et al., 2020, p. 973), the incentive behind the opening of new wards, rearrangement of spaces of care and ward assignments for COVID and non‐COVID patients. At the hospital where the nurse above works, nurses established a coordination system and cordoned off certain wards so as to prevent COVID patients from infecting others. They mobilised gatherings between materialities (the architectural environment and tarpaulin), the social (working routines and division of labour) and technologies (walkie‐talkies, personal protective equipment and COVID tests) in an effort to continue caring for patients. Such tinkering and acting upon sociotechnical assemblages to rearrange spaces of care are central to many of the cases in our data.

The prominent role of materialities and the built environment in reorganising spaces of care is illustrated in the following quote:

So, the doors of the isolation rooms are closed, without any windows. Well, then you can’t see the patients. But they were very sick, you want to be able to see them all the time and watch them through a window, and not have to put on a *[protective]* suit every time. We had those windows made immediately.

(Nurse, June 2020)

Having windows installed in the isolation rooms’ doors acted on the immediate need to observe patients closely without being in direct physical contact with them. This example illustrates how nurses experienced a mismatch between the unfolding needs of COVID treatment and established care practices in the built hospital environment. Hospitalised COVID‐19 patients require close observation, as they run a high risk of sudden deterioration and critical disease (Cecconi et al., 2020). However, at the same time, severe resource shortages required nurses to economise as much as possible on personal protective equipment (PPE), time and energy. Wearing protective clothing is also physically demanding and can be very uncomfortable (many nurses complained about headaches):

It was so hot working in protective clothing that we quickly asked for *[thinner]* surgical suits, but we never got them because they weren’t available. We kept working on COVID wards for entire shifts while dressed in protective clothing, wearing face masks, glasses, and gloves. Our glasses were all fogged up while we were puncturing IVs and distributing medication.

(Nurse, July 2020)

The new windows are an example of how nurses reassembled their built working environment to enable pandemic care delivery (Driessen, 2020; Mol et al., 2010). It also allowed nurses to limit direct patient contact, thereby also avoiding significant risks to their own health. This example also shows how they gained influence during the first wave. It has often been argued that nurses lack influence, control and agency over the organisation of care (Allen, 2014). In the tumult and extreme uncertainty of the pandemic, however, their requests were quickly met (e.g., other nurses mentioned how long wished‐for and expensive safety monitoring systems now arrived within a few days). This exemplifies a general observation in our study, which is that established and layered governance systems were, temporarily, breached early in the pandemic, making it possible for nurses to engage in organisational decision‐making and pursue organisational changes (i.e., architectural adjustments or more resources, staff and equipment).

In the following quote, a nurse reveals how spaces of care and routines were reassembled and how this involved different valuations:

We decided that our wards would be used only for patients with an “ICU plus policy” *[i.e., who would not be sent to the ICU if their clinical condition deteriorated].* We moved those patients, who most likely would not be treated in the ICU, to another unit. We did this so that we could distinguish better between patient flows. That worked quite well. But at the same time, you had to drop your work suddenly and move patients ad hoc who… well, I can remember one patient who was dying and had to be moved to another ward where I later thought, should we have done that?

(Nurse, September 2020)

This quote demonstrates how decision‐making is accompanied by high levels of uncertainty and organisational pressure. The upsurge of COVID patients forced nurses in this hospital to alter the normal coordination of patient flows. The ethical implications expressed by this nurse reveal the normativities involved in ‘doing’ COVID care. It shows the sometimes competing notions of good care that informed nurses’ daily crisis work, and how nurses navigated potential value conflicts without knowing the consequences (Latimer, 2018; Pols, 2014). In the crisis, nurses needed to prioritise what mattered most in a specific situation without being able to fall back on predetermined courses of action, and to mediate between the immediate and urgent needs of an individual patient and broader organisational and societal goals and values. In doing so, nurses weighed several organisational affordances, such as staff availability, changing care intensities and hospital admission rates. This tinkering with both collective and individual patient needs is a central observation in our study, which we will elaborate in the following sections.

### Coordination of care across hospital organisational boundaries

It was chaotic and panicky at the ICU. To ease pressure on staff, we decided to set up a buddy system. Some argued that the buddies should be nurses, while others opted for physicians. In the beginning, the buddies were nurses, but the ICU staff was not satisfied. They were quickly replaced by physicians. However, this presented yet another challenge. For example, physicians were asked to assist patients with their personal hygiene, with washing. Well, they said “yes, it’s done” but actually they had no idea. In the end, it turned out that some patients had not been washed for five days because the physician did not know how to do it. Or that they had brushed patients’ teeth with chlorhexidine.

(Nurse, November 2020)

The troubles accompanying the establishment of a buddy system reveal how patient and staff coordination was disrupted and needed to be reinvented. The reorganisation of COVID care involved not only epidemiological and clinical unknowns but also revealed that care coordination and care organisation were uncharted territory for health‐care workers, in this case physicians who lacked basic skills needed to perform daily hygiene care. In this section, we discuss and unravel nurses’ organising work by showing how they allocate and organise staff, teams, resources and working routines and methods within and across hospital departments. The next quote illustrates how the coordination of care involved decision‐making in situations of critical uncertainty:

When the first cohort unit was set up, we monitored it closely. How would it all work? At that time, we didn’t really know anything and needed to figure out how to organise care for COVID patients. One question was, for example, how do we organise food and nutrition services on these units? We were just constantly working on solving problems, setting up new units, and coordinating staff and materials.

(Nurse COVID coordinator, December 2020)

This nurse explains how staff, patients and resources needed to be re‐assembled and organised. Resource management involved situated experimentation and problem‐solving. This organising work was further intensified by local and global health‐care shortages (Juvet et al., 2021). Beyond the examples that attracted considerable media attention, such as the redistribution of ventilators and surgical masks, this nurse explains that the reorganisation of care involves many more aspects of daily care, for example, coordinating food and nutrition services. In our study, nurses point out staff shortages as one of the main challenges, for example, in the following quote:

We were continuously confronted with all kinds of issues for which there was no answer yet. We didn’t know what we were facing, and it was a process of learning and discovering what we had to do. The biggest challenge we encountered was that COVID care required a lot of nursing capacity. So, we had to figure out how we could staff cohort units. That was a major task, the numbers were growing, and we had to rearrange teams on a vast scale and ask everyone to cooperate. We tried to relieve nurses as much as possible by establishing “combination departments”, where nurses work both in the COVID units and within their specialism. Also, we set out to keep the teams together as much as possible so that nurses could share their experiences.

(Nurse, March 2021)

This quote reflects how nurses’ organising work unfolded across departments in staff coordination and allocation. This nurse explains how she tried to staff cohort units while attending to various organisational, quality and safety issues as well as the nurses’ wellbeing. Efforts to keep teams together as much as possible are important not only for mutual emotional support but also because quality and safety are embedded within teams and informal working structures and care routines (Mesman, 2011). In our study, we noted several cases in which nurses expressed feelings of insecurity and anxiety about working with changing staff and in different departments, pointing out the consequences of working with and without embedded routines and methods:

We were experimenting, figuring out how to work with nurses from all different departments. We started working on a new kind of classification and established new “types” of nurses. For example, Type 1 is a nurse who can perform care coordination. Type 2 is a nurse who can provide COVID care independently. Type 3 is a nurse who still needs to settle in or to be trained, and Type 4 is a nurse who has never worked in COVID care. We started using this classification system because we noticed that everyone has their own routines and methods. For example, something as simple as how you keep track of a patient’s fluid balance. Some departments measured that at eight o’clock, and other departments at midnight.

(Nurse, November 2020)

The classification system was established to coordinate, differentiate and align different ‘types’ of nurses during the COVID‐19 upsurge. During the peak of the pandemic, COVID care required high levels of staff flexibility. The military, medical students and civilian nurses were brought in to ease the pressure on hospitals and the nurse workforce. In the cases we observed, team composition varied greatly and depended on local circumstances. The above‐mentioned system was used by nurses to onboard new staff, enhance interprofessional collaboration and streamline working methods and routines. These seemingly mundane tools make visible how nurses deal with perceived hindrances in health‐care delivery and come up with temporal workarounds to manage care work in the crisis (Debono et al., 2013). Such interventions are critical, as several nurses mentioned a decline in the quality of nursing care due to the absence of embedded routines and methods and the changing teams:

Our team often claims that the crisis was not at the expense of quality of care. But that is absolutely not true. I think we can hardly remember what the care we provided a year ago looked like. We are constantly working with people from different departments, and that causes problems. For example, the double‐checking of medication. We do that in a computer system. However, not everyone understands this system. Recently a colleague asked me to double‐check the medication he had prepared. Out of six syringes, four were wrong! In this case, the mistake was averted, but in other cases, and during all the commotion, those syringes were simply used.

(Nurse, March 2021)

The examples in this section illustrate how nurses’ organising work goes beyond individual care trajectories and occurs across hospital departments. Central to the organising work of nurses is their ability to re‐assemble nurse teams and routines so as to enable pandemic care delivery, especially when the demand for care surges. In this process, nurses mediate between different values and ideas of good care (e.g., keeping care accessible for as many patients as possible while letting go of patient‐centred care delivery and knowing that mistakes could be, and were being, made). In the next section, we turn to how nurses operated within the broader crisis organisation and health‐care system to organise pandemic care.

### Care with the allocation algorithm

Tim chairs the meeting and asks about the current situation: “How many beds are available? How much capacity is there in the isolation rooms? How many unconfirmed patients are on the different nursing wards?” Nurses from different departments call out their numbers, some from memory, others reading the numbers from a sheet. “I’ve discharged five and have eight beds occupied”, one of the nurses replies. “We have no beds left and have already announced an admissions freeze”, another nurse responds. “So did we! We also have a patient who tested negative, we have cleared the isolation room. I now have eight in and four out”, yet another nurse adds. Tim writes the numbers down while an ICU nurse has the last word: “We can’t discharge anyone to the cohort unit today, and we don’t have any beds available, all patients are still in prone position”.

(Fieldnotes, November 2020)

This excerpt was taken from a hospital’s daily crisis meeting. It reflects how hospital capacity is coordinated and how the virus has driven Dutch hospitals to the limits of their capacity. However, the timing and impact of the virus varied across different regions and hospitals. In the early pandemic, some regions, and most major cities, experienced high rates of infection, while other regions had lower infection rates. Infection rates continued to fluctuate well into the subsequent waves of the virus. To distribute the care burden and, later in the pandemic, to safeguard non‐COVID care, a national coordination centre (the National Centre for the Spread of Patients, or LCPS) was established. The LCPS collects data on hospital capacity, regional infection rates, available ICU and other beds and hospital admissions. This information is entered into a digital system and processed into models, prognoses and scenarios that allocate patients equally. The Dutch Medical Association has drawn up the selection criteria for allocation, and the numbers are presented in daily spreadsheets and circulated several times a day (Wallenburg et al., 2021). In the Dutch COVID‐19 response, the LCPS became an important new actor in the health‐care system, and the algorithm underpinning its predictions became a key tool. Nurses played an important role in working with the algorithm and coordinating patient allocation. We use the case of patient allocation to demonstrate how the organising work of nurses contributes to the national transfer system by translating the algorithmic decisions into real‐life reallocation, bringing care into the crisis‐transfer system:

It’s good that we *[allocation coordinators]* are nurses. Physicians have medical expertise but have no idea how the clinic works. We have broad clinical knowledge but are also knowledgeable about what it entails and understand the whole picture.

(Nurse coordinator, November 2020)

This nurse explains how she and her colleagues consider ‘the whole picture’ when reallocating patients. Nurses are often referred to as ‘the glue in the system’, the passage points in hospitals, mediating relations between dispersed actors and synthesising the clinical, practical and organisational knowledge of various departments and patients’ psychosocial circumstances (Allen, 2014). This nurse’s argument is echoed in other cases in our study, complementing the critical role of nurses in coordinating patient allocation. In the Dutch context, this also involved managing patient transfers to other care facilities, such as nursing homes and rehabilitation care centres. The next quote shows a nurse COVID coordinator working on patient allocation:

Tim views the two new files. The first file appears to be in order, but the second file raises questions. Tim notes that the patient’s partner has also been admitted to hospital. He picks up his phone and calls the department: “Isn’t this a bit sad? I mean, have you reviewed it?” At the other end of the line, the nurse enquires and says: “No, the patient doesn’t think it’s a problem”. Tim rolls his eyes and looks at me: “Okay, well then we’ll register him after all”. He starts entering the patient’s details in the system, but suddenly stops and reaches for his phone again: “I just think it’s a bit inappropriate, is there really no other option? I just want to double‐check; I also see that there is already something about aftercare in the file. Isn’t the patient about to go home?” Tim turns his chair to the window and waits for an answer. After minute or two, Tim is informed that the patient can stay in hospital. Tim puts his phone down and says to me: “I didn’t like it, it’s just not friendly, the oxygen level is okay now, and she hasn’t been given any supplemental oxygen at all”.

(Nurse and COVID coordinator, November 2020)

The negotiation described above shows the work needed to translate and do the reallocation work in practice and how nurses are normatively engaged. The nurse in question works with a centralised matching system established by the LCPS in which COVID coordinators register patients for transfers. The system, in turn, shows which hospitals have open beds. When there is a match, COVID coordinators are contacted by LCPS health‐care workers to manage the transfers. The LCPS then contacts the receiving hospitals and sends ambulances for transfers.

The above excerpt describes how the nurse tries to prevent the transfer of a patient whose partner is also hospitalised. It shows the nurse using different types of knowledge—for example, of clinical needs (medical records, COVID‐19 clinical features), of the organisation (after‐care, care trajectories, allocation process) and of the patients’ psychosocial circumstances (the patient’s wife is also hospitalised)—to figure out the ‘right’ place for this patient. This nurse hence mediates between different health‐care actors to repair and adjust the outcome of the algorithm and articulates a form of patient‐centred care. He does so despite the models and algorithm showing that there is little capacity left and that the patient meets the criteria for transfer.

We see here that the algorithms and LCPS models are not stable systems but involve hands‐on professional work and continuous maintenance, repair and translation (Denis & Pontille, 2017). This example and many more in our data show how nurses using the allocation algorithm continuously navigate individual and collective needs and in doing so weigh up variable capacity within and across organisations, patients’ socio‐psychological circumstances, clinical features and the ins and outs of various hospital departments in order to assess whether a patient should—or should not—be transferred.

The next step in coordinating patient allocation is the actual transfer of patients. In the following quote, we see how a nurse at the LCPS manages transfer logistics and coordinates ambulances for transfers:

Stef shows me a file in the system and says: “This patient has had a brain haemorrhage, has COVID, and is registered for transfer. It is a complex case, which makes me think right away, what is important here? What is his neurological condition, does he have trouble swallowing, can he speak well, and how much does he weigh? Those are important questions because we need to provide a good and humane transfer and *[ambulance]* ride. I am the last checkpoint here, and I try to understand the broader clinical picture, not just COVID. That is essential for the type of ambulance we deploy”.

(Nurse, February 2021)

The data entered into the LCPS system determines whether a regular ambulance or a mobile ICU ambulance is called in to transfer patients. However, the above quote points out how ambulance coordination also involves critical articulation work (Star & Strauss, 1999). It reflects how this nurse validates and interprets different types of information (i.e., clinical, organisational, practical) to determine which type of ambulance is suitable for this patient’s transfer. We see how the nurse assesses the patients’ clinical condition and connects this with available sociotechnical affordances and, in doing so, articulates a form of ‘good and humane’ transfer care.

These examples further underscore how nurses set out to and succeed at influencing the organisation of crisis care and how they perform organising work across organisational boundaries and for the health‐care system. As the next quote shows, however, this work is not always acknowledged and appreciated in nursing practice. In our study, several nurses said that they felt their broader organising work went unappreciated, both by policymakers and managers, but also within teams and among nurses themselves:

Nobody on my own team said: you’re doing a good job. No one!

(Nurse, March 2021)

This perceived lack of appreciation from peers is notable but unsurprising in light of the invisibility of nursing work. Nursing work is articulation work; the work that is needed ‘to get things back on track’ (Star & Strauss, 1999), and that is played out at the patient’s bedside or, in this case, behind a desk to coordinate an ambulance ride. It is work that is often unseen or unacknowledged. Nurses complain about this lack of public recognition—but also the lack of recognition from their nursing colleagues.

The examples in this final empirical section show how nurses play a critical role in allocating patients, and how different perspectives on good care are coordinated to accomplish ‘good’ patient transfers. They reveal how nurses make the algorithm and allocation work concrete and applicable in daily crisis care and play an important role in coordinating patient transfers and in motivating, strategising and convincing other health‐care professionals to transfer or to not transfer a patient. In doing so, nurses play a crucial role in maintaining and organising patient allocation in an overburdened system.

## DISCUSSION

Nurses have played (and are still playing) a crucial role in pandemic care delivery, not only by caring for severely ill patients but also by keeping the organisation of care and the broader health‐care system on track. In this article, we show how nurses accomplish this by assembling organisational, material and clinical knowledge as well as using their in‐depth understanding of patients’ psychosocial circumstances in their care work. We demonstrate how nurses act upon and organise the built environments in which COVID care is performed, and how they reinvent working methods and routines across hospital departments (e.g., by establishing temporal classifications, putting together ‘combination teams’ and allocating resources across wards). Empirical data on the allocation of COVID patients furthermore shows how nurses, as central actors in dispersed and fragmented hospital care, organise and perform patient allocation across organisations. These insights help to disclose how nurses actively mobilise and renegotiate the allocation algorithm and mediate different valued purposes (i.e., patient‐centred care, timely care, the needs of the whole population) and connect this with the sociotechnical affordances of hospital and other care (e.g., daily rhythms of the clinic, hospital capacity, staff and resource availability).

In answering our main research question, that is, how nurses assemble or reassemble care at different organisational levels in the Dutch COVID‐19 response, we show in our analysis how nurses are experimentally inventing COVID care along the way and performing, translating and rendering ‘ideas on paper’ applicable in daily care work. Nurses are knowledgeable and flexible organisers of care, mediating, bringing together and mobilising heterogeneous elements (e.g., people, concepts, things and technologies) in their real‐world practice. By moving between different layers of the health‐care system, we demonstrate how nurses’ organising and care work plays out and connects the patient, the family, the hospital and the broader crisis organisation and health‐care system.

This study furthermore articulates how, in the COVID‐19 pandemic, nursing work involves continuously tinkering with both individual and collective patient needs and the inherent normativities. In line with previous studies, we reveal how care involves situated and experimental tinkering (Mol et al., 2010) to bring together conflicting values (i.e., transferring a dying patient to make room for new infected patients in hospital). In the analysis, we outline how different values and goods, namely care for the individual, the organisation and broader society, inform nurses’ daily practice in the pandemic. Our findings suggest that these values and normativities are highly situated and far from stable, and that tinkering with different values mediates between specific contexts, histories and nurses’ understanding of patients’ psychosocial circumstances and hospitals’ sociotechnical affordances. Moreover, we demonstrate how notions of safety and quality are segmented and translated in times of crisis (e.g., by [not] ensuring safe medication dosing by double‐checking, provisional implementation of buddy systems) and involve a constant weighing of what matters most and balancing of different valuations in constrained circumstances (‘good’ care or ‘good enough’ care, ‘safety’ or ‘accessibility’).

These findings put forward an alternative understanding and conceptualisation of nurses and nursing work. This is important because, in the words of Nassim Nicholas Taleb, ‘Ideas come and go. But stories stay’ (2007, p. 26). While the ‘nurses as heroes discourse’ frames nurses as compassionate helpers and confirms public images of nursing as primarily a hands‐on and care‐giving profession at the bedside, our findings reveal that nurses are active and critical actors in the COVID‐19 response. We have used the metaphor of uncharted territory to describe the many unknowns, unpredictability and uncertainties nurses are dealing with in the COVID crisis and through their organising work. Sticking with this metaphor, we argue that nurses in the Dutch COVID‐19 response can be seen as explorers who renegotiate professional standards and guidelines, examining unfamiliar terrain with their experimental and reflexive practices and learning to deal with a new disease and new organisational circumstances. As illustrated in the opening quote of this article, both managers and physicians recourse to nurses’ explorative work to lead the way in the COVID crisis.

The sociological literature on health‐care organisations generally ignores nurses’ organising work, but that work is also unseen and unappreciated in daily health‐care practice. It took months before nurses were added to crisis management teams in Dutch hospitals and only in the second COVID‐19 wave were they invited to crisis meetings and allowed to contribute to organisational decision‐making. Unfortunately, this broader appreciation of nurses’ organisational competencies appears to have been short‐lived. While staff shortages are growing, nurses still appear to be struggling to position themselves as critical and active organising agents in the COVID‐19 response. This is further complicated by a powerful counterforce within the nursing profession itself that values patient work above organisational work (Felder et al., 2022). It is therefore important to add a nursing political and organisational discourse to the dominant microlevel care discourse (Tronto, 1993). The framing of nurses as heroes is increasingly at odds with frontline nursing work in the COVID‐19 pandemic. We argue that our conceptualisation of nurses as explorers who mediate and move between different layers of the health‐care system, thereby assembling and reassembling care at different organisational levels, calls for an alternative acknowledgment and appreciation of nursing work in the crisis and beyond.

## AUTHOR CONTRIBUTIONS

**Martijn Felder**: conceptualisation (equal); formal analysis (supporting); project administration (equal); supervision (equal); writing—original draft (supporting). **Roland Bal**: conceptualisation (equal); formal analysis (supporting); project administration (equal); supervision (equal); writing—original draft (supporting). **Iris Wallenburg**: conceptualisation (equal); formal analysis (supporting); project administration (equal); supervision (equal); writing—original draft (supporting).

## CONFLICT OF INTEREST

The authors declare that there is no conflict of interest that could be perceived as prejudicing the impartiality of the research reported.

## Data Availability

The data that support the findings of this study are available on request from the corresponding author. The data are not publicly available due to privacy or ethical restrictions.
